# Sitagliptin and the Blood-Retina Barrier: Effects on Retinal Endothelial Cells Manifested Only after Prolonged Exposure

**DOI:** 10.1155/2020/2450781

**Published:** 2020-05-25

**Authors:** Anja Jäckle, Focke Ziemssen, Eva-Maria Kuhn, Jürgen Kampmeier, Gerhard K. Lang, Gabriele E. Lang, Helmut Deissler, Heidrun L. Deissler

**Affiliations:** ^1^Department of Ophthalmology, University of Ulm, Prittwitzstrasse 43, 89075 Ulm, Germany; ^2^Centre of Ophthalmology, Eberhard Karls University Tübingen, Elfriede-Aulhorn-Straße 7, 72076 Tübingen, Germany; ^3^Department of Obstetrics and Gynecology, University of Ulm, Frauensteige 14, 89075 Ulm, Germany; ^4^HD/U, 89075 Ulm, Germany

## Abstract

Inhibitors of dipeptidyl peptidase-4 (DPP-4) are widely used to treat diabetes mellitus, but data concerning their effects on the barrier stability of retinal endothelial cells (REC) in vivo and in vitro are inconsistent. Therefore, we studied whether the barrier properties of immortalized endothelial cells of the bovine retina (iBREC) were affected by the inhibitors of DPP-4 sitagliptin (10-1000 nM) and diprotin A (1-25 *μ*M). Their effects were also investigated in the presence of VEGF-A_165_ because diabetic patients often develop macular edema caused by VEGF-A-induced permeability of REC. To detect even transient or subtle changes of paracellular and transcellular flow as well as adhesion of the cells to the extracellular matrix, we continuously monitored the cell index (CI) of confluent iBREC grown on gold electrodes. Initially, the CI remained stable but started to decline significantly and persistently at 40 h or 55 h after addition of sitagliptin or diprotin A, respectively. Both inhibitors did not modulate, prevent, or revert the persistent VEGF-A_165_-induced reduction of the CI. Interestingly, sitagliptin and diprotin A increased the expression of the tight-junction protein claudin-1 which is an important component of a functional barrier formed by iBREC. In contrast, expressions of CD29—a subunit of the fibronectin receptor—or of the tetraspanin CD9 were lower after extended treatment with the DPP-4 inhibitors; less of the CD9 was seen at the plasma membrane after prolonged exposure to sitagliptin. Because both associated proteins are important for adhesion of iBREC to the extracellular matrix, the observed low CI might be caused by weakened attachment of the cells. From our results, we conclude that extended inhibition of DPP-4 destabilizes the barrier formed by microvascular REC and that DPP-4 inhibitors like sitagliptin do not counteract or enhance a VEGF-A_165_-induced barrier dysfunction as frequently observed in DME.

## 1. Introduction

Dipeptidyl peptidase-4 (DPP-4/CD26) cleaves a dipeptide from the N-terminal part of proteins or peptides thereby inactivating most substrates which include chemokines, neuropeptides, and incretins like glucagon-like peptide-1/-2 (GLP-1/-2) [1]. In addition to its enzymatic activity, the DPP-4 homodimer also interacts and forms complexes with proteins involved in various cellular processes, e.g., cell adhesion to the extracellular matrix or transcellular transport [1–3].

DPP-4 inhibitors like the orally administered sitagliptin are widely used in the therapy of diabetes mellitus as they prolong the action of incretin hormones, including GLP-1 and glucose-dependent insulinotropic polypeptide, by interfering with their degradation in vivo [4–8]. Effects of DPP-4 inhibitors on the blood-retina barrier (BRB) in vivo however are still unclear: sitagliptin-treated diabetes type 2 patients in a cardiovascular outcome study (TECOS) slightly more frequently developed diabetic retinopathy than the placebo control group, but a systematic and standardized documentation of visual acuity and retinal findings was not made [9]. Initial concerns were, however, not confirmed by results of another study (NCT00838903) in which stable visual functions were observed.

The results of sitagliptin studies with cell culture and animal models were also inconsistent and therefore inconclusive [10–14]. An increased permeability of macrovascular human endothelial cells of the umbilical cord (HUVEC) was evident shortly after addition of DPP-4 inhibitors sitagliptin (0.1-10 *μ*M) or diprotin A (1-100 *μ*M), accompanied by relocalization of the adherens junction (AJ) protein vascular endothelial cadherin (VEcadherin) from the plasma membrane [10]. In contrast, 100 nM sitagliptin had no effect on the barrier formed by bovine retinal microvascular endothelial cells (BREC) during exposure for six hours, and expression of the tight junction (TJ) protein claudin-5 remained stable under these conditions [12]. Another inhibitor of DPP-4, linagliptin, prevented TNF*α*-induced disturbances of human retinal endothelial cells (huREC) and interfered with the interaction of monocytes with the endothelial cells [13].

Elevated permeability of retinal EC (REC) observed in vision-threatening diabetic macular edema (DME) mostly results from increased expression of the growth factor VEGF-A in the vitreous [15, 16]. Permeability of monolayers of primary or immortalized REC isolated from various species can be induced by growth factors like VEGF-A or TNF*α*, and it correlates with changes of the compositions of TJ and AJ, the complexes regulating paracellular flow. In these experiments, observed changes were dependent on duration of treatment and the growth factor(s) used [17–20]. Slightly decreased amounts of the AJ protein VEcadherin at the plasma membrane of (human) REC were observed during the first few hours after addition of VEGF-A, whereas extended treatment of immortalized BREC (iBREC) or huREC with this growth factor for up to three days resulted in the loss of the TJ protein claudin-1 [18–23]. This was accompanied by subtle changes of the plasma membrane localizations of claudin-5 and VEcadherin, and caveolin-1—involved in transcellular transport—was not affected [21–23]. However, after treatment of BREC for several hours with TNF*α*, claudin-5 had disappeared from the plasma membrane [12].

In view of the inconsistent results of already published studies regarding this question, this investigation was initiated to clarify how the barrier function of REC might be affected by prolonged treatment with the structurally unrelated DPP-4 inhibitors sitagliptin or diprotin A for several days. As an appropriate and well-established model, we chose immortalized BREC which have been used for many years to predict changes induced by various effectors relevant to genesis and progression of DME [18, 23–26]. To determine even subtle and transient effects of the DPP-4 inhibitors on an iBREC monolayer noninvasively during prolonged cultivation over several days, we performed continuous electric cell-substrate impedance measurements with a microelectronic biosensor system for cell-based assays [22, 27–29]. Changes of the measured impedance can be caused by different compositions of the TJ- (containing claudin-1 and claudin-5) or AJ- (with VEcadherin) regulating paracellular flow, enhanced transcellular transport (involving caveolin-1), or impaired adhesion to the substrate (e.g., mediated by CD9 and CD29) [18–22, 27–30]. As part of a more comprehensive approach, expression, and subcellular localizations of the above mentioned, potentially affected proteins were assessed.

## 2. Materials and Methods

### 2.1. Reagents and Antibodies

Information about the antibodies and inhibitors used in this study is provided in Tables 1 and 2, respectively. All inhibitors were dissolved in dimethyl sulfoxide (DMSO; Sigma-Aldrich, Steinheim, Germany) to result in final solvent concentrations below 0.05% in the culture medium which did not affect the morphology or behavior of iBREC. Recombinant human *Sf*21-expressed VEGF-A_165_ (293VE) was purchased from Bio-Techne (Wiesbaden, Germany).

### 2.2. Cultivation of iBREC

Generation and characterization of telomerase-immortalized microvascular endothelial cells from bovine retina (iBREC) have been described in detail [23]. We used these cells between passages 25 and 55 counting from the stage of primary culture for which we confirmed stable expression not only of investigated proteins, e.g., DPP-4, TJ and AJ proteins, CD9, and CD29, but also of marker proteins characteristic of EC [21, 23–25]. In contrast to primary REC, cultures of iBREC were completely free of *α*-smooth muscle actin-expressing cells [23]. As an additional quality control to confirm authenticity and stability of the cells, the characteristic proliferation profile of iBREC was routinely recorded by electric cell-substrate impedance measurements (see Section 2.5) [22]. We cultivated iBREC in Endothelial Cell Growth Medium MV (ECGM; PromoCell, Heidelberg, Germany) containing 1 g/l glucose, 0.4% Endothelial Cell Growth Supplement/H, 90 *μ*g/ml heparin, 10 ng/ml human epidermal growth factor (hEGF), 100 nM hydrocortisone, 5% fetal bovine serum (FBS; all supplements were from PromoCell), and 0.3 mg/ml geneticin (Thermo Fisher Scientific) always on fibronectin-coated (Corning, Amsterdam, the Netherlands) surfaces until a confluent monolayer was formed after three to four days [21, 23–25].

### 2.3. Concentrations of Active Substances

**Figure 1:**
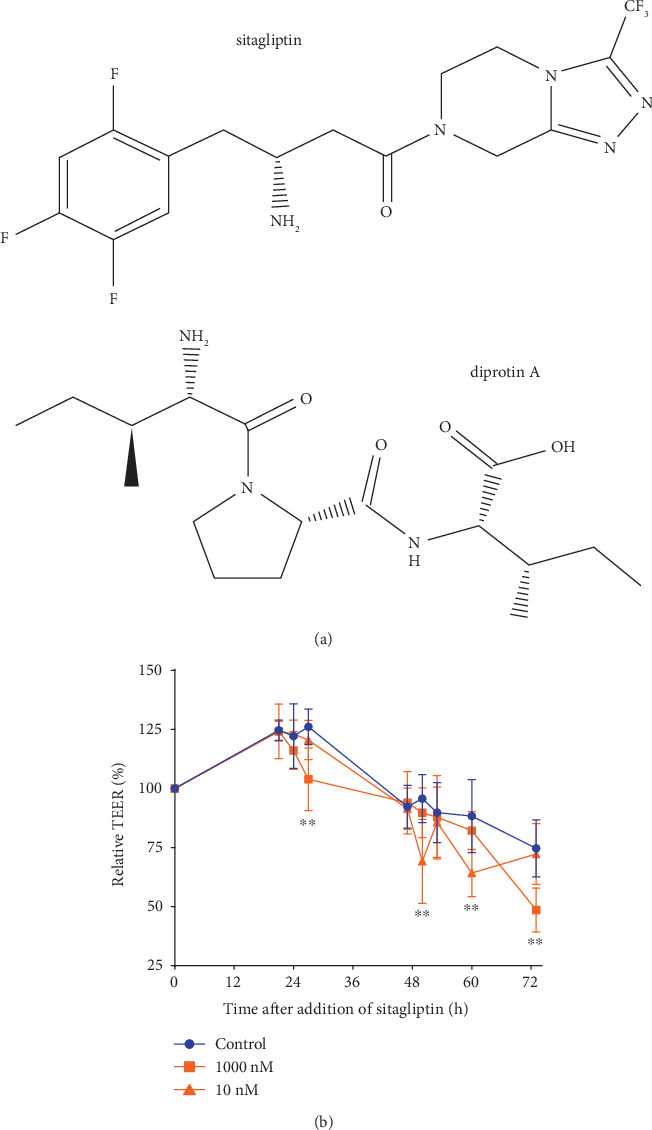
Prolonged treatment with sitagliptin only weakly changed the TEER of unchallenged iBREC. (a) Structures of DPP-4 inhibitors used. (b) Confluent iBREC grown on porous membrane inserts were exposed to 10 or 1000 nM sitagliptin for up to three days. The transendothelial electrical resistance (TEER), determined as a measure of permeability at indicated time points, was only weakly and inconsistently reduced by the inhibitor. TEER values, normalized in relation to those measured immediately before addition of sitagliptin, are shown as means and standard deviations of data from at least four replicates. Statistical analyses were performed as described in Materials and Methods. ^∗∗^*p* < 0.01 compared to control.

In all experiments, inhibitors of the DPP-4 (sitagliptin and diprotin A; structures are shown in Figure 1(a)) were used at concentrations close to their published IC_50_ values; a concentration of 1 *μ*M sitagliptin was included in the experiments because plasma levels of 1 *μ*M can be reached by administration of an oral dose of 100 mg sitagliptin once daily; higher concentrations were not tested in view of potential unspecific effects [4, 5, 31].

### 2.4. Treatment of iBREC with Sitagliptin and Measurements of Transendothelial Electrical Resistances

iBREC (5 × 10^3^) were seeded on fibronectin-coated membrane inserts (Transwell permeable supports, 0.33 cm^2^ polyester membrane, pore size 0.4 *μ*m, 4 × 10^6^ pores/cm^2^, Corning) and cultivated in ECGM until confluence was reached three to four days later. The culture medium was then replaced by serum-reduced culture medium (SRM; same as ECGM but without hEGF and containing 0.25% FBS and 1 *μ*g/ml fibronectin) for one day before sitagliptin (final concentrations: 10 nM or 1000 nM) was added to both chambers for an additional three days. Transendothelial electrical resistances (TEER) across the cell layers were measured with hand-held chop stick electrodes and a Millicell ERS resistance meter (Millipore, Schwalbach, Germany) at indicated time points [18, 21, 25, 32]. To avoid temperature-induced changes in the TEER, plates were kept on a warm plate at 37°C during measurements [33]. Normalized TEER values (*n* ≥ 4 for each condition) were calculated in relation to those measured in SRM just before addition of effectors.

### 2.5. Treatment of iBREC with Effectors and Cell Index Measurement

We also assessed the barrier stability of iBREC cultivated on gold electrodes by performing continuous electric cell-substrate impedance measurements with the microelectronic biosensor systems for cell-based assays xCELLigence RTCA DP (Acea, OLS, Bremen, Germany) as previously described: ~10^4^ cells were seeded per fibronectin-coated well of an E-Plate 16 PET (Acea); impedance was measured between gold electrodes in each individual well and expressed as the unit-free parameter cell index CI = (*Z*_*i*_ − *Z*_0_)/15 *Ω* (RTCA Software 2.0, Acea) [22, 27]. In this formula *Z*_*i*_ is the impedance measured at an individual time point and *Z*_0_ the impedance read at the start of the experiment [27]. After a confluent cell monolayer had been formed within three to four days indicated by a high cell index (CI ≈ 18), the culture medium was replaced by 180 *μ*l SHM (same as ECGM but without hEGF and containing 1 *μ*g/ml fibronectin) and the CI was measured every 15 min for one day. Effectors (i.e., sitagliptin, diprotin A, or VEGF-A_165_ or a combination thereof; final concentrations are listed in Table 2) were then added in 20 *μ*l basal medium (BM; same as ECGM but without hEGF, FBS, and hydrocortisone and containing 1 *μ*g/ml fibronectin), and CI was subsequently determined every 2 min for 2 h, followed by measurements every 5 min for up to three days. To study whether tivozanib or sitagliptin reverted or otherwise modified the VEGF-A-induced decline of the CI, confluent iBREC cultivated in 180 *μ*l SHM for one day were exposed to VEGF-A_165_ (final concentration: 50 ng/ml; added in 10 *μ*l BM) for one day (CI measurements every 2 min for 2 h, then every 5 min) before inhibitors (in 10 *μ*l BM; final concentrations: see Table 2) were added. Again, the CI was measured every 2 min for 2 h and then every 5 min for up to two days. Recorded CI values (*n* ≥ 6 for each condition and time point) were normalized in relation to those measured immediately before addition of effectors (RTCA Software 2.0), and the results were converted to graphs showing means and standard deviations with GraphPad Prism 6 (GraphPad Software, San Diego, USA) [22].

### 2.6. Immunofluorescence Stainings, Preparation of Protein Extracts, and Western Blot Analyses

Confluent monolayers of iBREC were exposed to sitagliptin (final concentrations: 10-000 nM) or diprotin A (final concentrations: 1-25 *μ*M) for two or three days, respectively, before they were either fixated for immunofluorescence stainings or harvested for preparations and analyses of protein-containing extracts. Immunofluorescence staining of confluent iBREC cultivated on two-chamber slides (x-well PCA Tissue Culture Chambers; Sarstedt, Nuembrecht, Germany) was performed to determine subcellular localizations of proteins of interest as described in detail elsewhere [22, 34]. To detect antibody-specific signals, Alexa Fluor 595-conjugated goat anti-rabbit secondary F(ab′)_2_ fragments (*λ*_ex_/*λ*_em_ = 596 nm/620 nm; A11072; Thermo Fisher Scientific) diluted 1 : 500 in 1% ImmunoBlock/PBS (Carl Roth, Karlsruhe, Germany) were used. Slides were embedded in ProLong Gold/Diamond Antifade Mountant (P36935; Thermo Fisher Scientific) with 4′,6-diamidino-2-phenylindole (DAPI; *λ*_ex_/*λ*_em_ = 359 nm/461 nm) for examination with a fluorescence microscope (model DM4000B, software FW4000, Leica, Wetzlar, Germany). DAPI-stained nuclei were counted in forty randomly chosen microscopic fields for assessment of a potential effect of sitagliptin on cell vitality.

For preparation of whole cell extracts, ~2 × 10^6^ iBREC cultivated in fibronectin-coated cell culture flasks (75 cm^2^, Sarstedt) were suspended in 300 *μ*l ice-cold lysis buffer 17 (Bio-Techne) supplemented with 5 *μ*g/ml aprotinin, 10 *μ*g/ml pepstatin, 10 *μ*g/ml leupeptin (all Roche Diagnostics, Mannheim, Germany), and phosphatase inhibitor cocktail 2 (1 : 200; P5726, Sigma-Aldrich, Steinheim, Germany), and the suspension was incubated on ice for 30 min under gentle agitation. After subsequent centrifugation (21100 × g for 20 min at 4°C) the resulting supernatant was frozen and stored at -80°C. Proteins of interest present in the extracts were determined by Western blot analyses as described previously [22]. Usually, protein samples were separated under reducing conditions, but to detect signals specific for CD9 or CD29, samples were analyzed under nonreducing conditions. Chemiluminescence signals were visualized by direct scanning with the imaging system Fusion Pulse TS (Vilber Lourmat, VWR, Darmstadt, Germany); peak volumes of the corresponding bands were determined with Evolution Capt software (Vilber Lourmat) and standardized in relation to those of actin from the same sample. To compare independent experiments, values were normalized in relation to those obtained from experiments with control cells processed in the same way without effectors.

### 2.7. Immunoprecipitation and Far-Western Blot

To prepare whole cell extracts for immunoprecipitation, 2 × 10^6^ iBREC were detached by scraping, resuspended in 300 *μ*l cold lysis buffer (40 mM TrisCl, 150 mM NaCl, 1% Brij 97 (Sigma-Aldrich), pH 7.4, supplemented with the EDTA-free protease inhibitor cocktail (Roche Diagnostics)), and incubated on ice for 1 hour. The supernatant resulting from subsequent centrifugation (15 min at 21100 × g, 4°C) was stored at -80°C [30]. These cell extracts were incubated with antibodies specific for CD9 or CD29 or an isotype-matched control IgG (final antibody concentrations: 10 *μ*g/ml) under gentle agitation on ice for one hour. To precipitate formed protein-antibody complexes, Protein G Plus/Protein A-Agarose beads (15 *μ*l per 1 *μ*g antibody; Calbiochem/Merck, Darmstadt, Germany) were added. After incubation for one hour under constant agitation at 4°C, beads were separated by centrifugation (3 min for 200 × g) and washed three times with lysis buffer. Bound antibodies—free or attached to the targeted proteins—were removed from the beads by incubation with 1% sodium dodecyl sulfate (*w*/*v*) for 10 min at 60°C. Immunoprecipitates and whole cell extracts were analyzed by Western blot, and X-ray films (Hyperfilm ECL, GE Healthcare, VWR) were exposed to the resulting chemiluminescence (SuperSignal® Substrate kit; Thermo Fisher Scientific) for visualization of signals.

### 2.8. Statistical Analyses

One-way analyses of variance (ANOVA)—followed by Tukey's range test to find differing groups—were applied (GraphPad Prism 6) to compare measured CI values at selected time points or quantified antigen-specific signals from Western blot analyses. The nonparametric Wilcoxon signed rank test was used to compare antigen-specific Western blot signals from effector-treated cells to the hypothetical value of 1.0 of normalized signals from control cells. To compare measured TEER values, the two-way ANOVA—followed by Tukey's range test to find differing groups—was applied. Differences resulting in *p* values below 0.05 were considered significant. In addition to providing means and corresponding standard deviations, results were presented as scatter plots including these values. All experiments were repeated at least twice.

## 3. Results

### 3.1. Sitagliptin Persistently Decreased the Cell Index of Unchallenged iBREC

Increased paracellular and/or transcellular flow is indicative of a higher EC barrier permeability and correlates with a decreased transendothelial electrical resistance (TEER) of the cell monolayer [32]. When confluent iBREC monolayers grown on porous membrane inserts had been exposed to 10 nM or 1 *μ*M sitagliptin for up to three days, the measured TEER was only weakly changed: compared to control cells, the TEER of the sitagliptin-treated cells was only slightly lower after 40 h (10 nM sitagliptin) or 72 h (1 *μ*M sitagliptin) of exposure (Figure 1(b)). Initially, four hours after addition of sitagliptin, the TEER was not affected compared to control cells (control: 75.6% ± 2%, 10 nM sitagliptin: 72.7% ± 20%, 1 *μ*M sitagliptin: 86.1% ± 10.6%; *p* > 0.05; values normalized in relation to those measured immediately before addition of sitagliptin).

**Figure 2:**
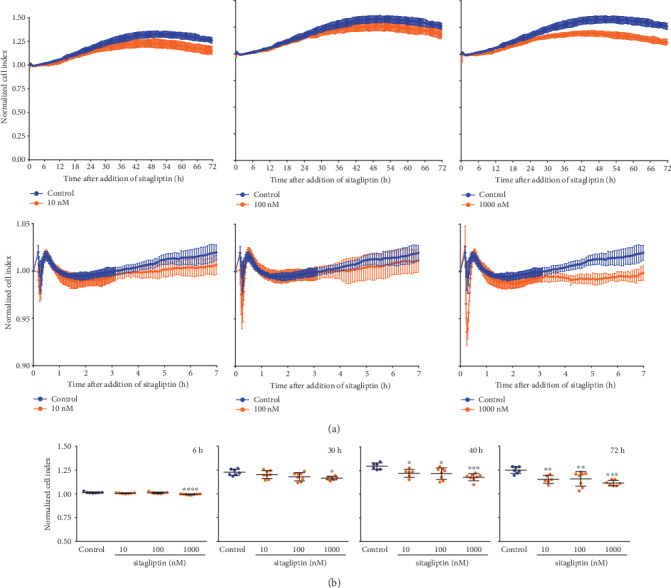
Treatment with sitagliptin reduced the cell index of unchallenged iBREC. Cells were cultivated on gold electrodes until confluency was reached and exposed to sitagliptin over three days. The cell index (CI) was determined continuously as a measure of barrier function. Sitagliptin (10-1000 nM) resulted in a persistent, concentration-dependent CI decline starting six to forty hours after addition. (a) CI values, normalized in relation to those measured immediately before addition of sitagliptin, are shown as means and standard deviations of data from at least five wells. (b) Statistical analyses of data gained at indicated time points after addition of sitagliptin were performed as described in Materials and Methods. ^∗^*p* < 0.05, ^∗∗^*p* < 0.01, ^∗∗∗^*p* < 0.001, and ^∗∗∗∗^*p* < 0.0001 compared to control.

To detect even subtle and transient changes associated with increased paracellular flow and/or transcellular transport or weaker adhesion of the cells, we continuously measured the cell index (CI) of iBREC cultivated on gold electrodes [22, 27, 28]. Exposure of the cells to 10 nM or 100 nM sitagliptin resulted in a significant and persistent decrease of the CI evident about 40 h after its addition (Figure 2). The effect of the highest tested sitagliptin concentration of 1 *μ*M was visible as early as six hours after it had been added (Figure 2). To rule out that observed effects were due to structural changes of the cell monolayer caused by cell loss or morphological conversion, we confirmed constant numbers of cells and their stability: nuclei of the fixed cells treated with 10-1000 nM sitagliptin for two days were counted, but their numbers were not different from those determined for control cells (100% ± 11.7%, 10 nM sitagliptin: 95.6% ± 10.3%, 100 nM sitagliptin: 95.1% ± 11.9%, 1 *μ*M sitagliptin: 93.6% ± 9.5%; *p* > 0.05, *n* = 40 for each condition). Also, we did not observe any effect on their morphology when iBREC were exposed to sitagliptin for several days.

### 3.2. Sitagliptin Did Not Change the Cell Index of VEGF-A_165_-Treated iBREC

**Figure 3:**
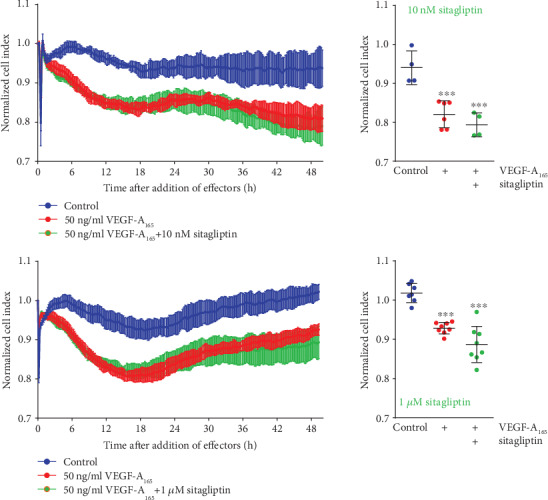
Sitagliptin did not modulate the barrier dysfunction induced by VEGF-A. Confluent monolayers of iBREC cultivated on gold electrodes were exposed to 10 or 1000 nM sitagliptin in combination with VEGF-A_165_ over two days. The VEGF-A_165_-induced decline of the CI was not affected by the DPP-4 inhibitor. Normalized CI values are presented as means and standard deviations from at least four wells. Normalization and statistical analyses of data recorded at 48 h after addition of sitagliptin and VEGF-A_165_ were performed as described in Materials and Methods. ^∗∗∗^*p* < 0.001 compared to control.

**Figure 4:**
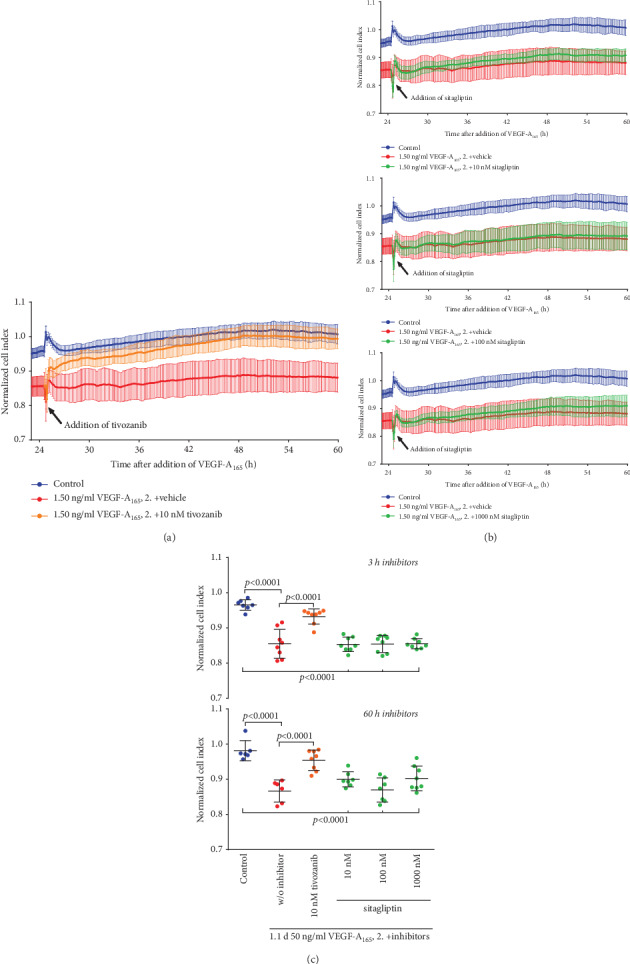
Tivozanib but not sitagliptin reverted the VEGF-A-induced barrier dysfunction of iBREC. Confluent monolayers of iBREC cultivated on gold electrodes were exposed to 50 ng/ml VEGF-A_165_ (*t* = 0 h) for one day before (a, c) 10 nM tivozanib or (b, c) 10-1000 nM sitagliptin was added (*t* ≈ 24 h). The cell index (CI) was determined continuously as a measure of barrier function. In all experiments, CI values—presented as means and standard deviations from at least six wells—were normalized in relation to those measured immediately before addition of VEGF-A_165_ (*t* = 0 h). Statistical analyses of data gained at indicated time points after addition of VEGF-A_165_ were performed as described in Materials and Methods. (a, c) Inhibition of the VEGF receptor 2 completely reverted the VEGF-A_165_-induced CI decrease. (b, c) Treatment with sitagliptin did not result even in partial reversion of the VEGF-A_165_-caused CI reduction.

Elevated permeability of REC induced by VEGF-A plays a dominant role in the development of DME [16]. Therefore, we investigated whether sitagliptin also modulated the VEGF-A-induced barrier dysfunction of iBREC. Treatment of a confluent iBREC monolayer with 50 ng/ml VEGF-A_165_ resulted in a stable and strong decrease of the CI apparent a few hours after its addition (Figure 3) which could be prevented by inhibition of VEGF receptor 2 with 10 nM tivozanib [22]. Inhibition of DPP-4 did not have a similar protecting effect on the CI-sensitive barrier function as shown by treating iBREC with both 50 ng/ml VEGF-A_165_ and sitagliptin (final concentrations: 10 nM and 1 *μ*M) for up to two days (Figure 3). To investigate whether the inhibitor of DPP-4 reverted the decline of the CI induced by VEGF-A_165_, cells were exposed to the growth factor for one day, before sitagliptin (final concentrations: 10-1000 nM) was added with VEGF-A_165_ still being present. We have previously shown that after exposure of iBREC to VEGF-A_165_ for one day, the dysfunction of the barrier formed by a monolayer of these cells is pronounced [18, 21, 22, 25]. However, sitagliptin was not able to counteract or even revert the VEGF-A_165_-induced decline of the CI (Figure 4(b)), in contrast to efficient normalization by 10 nM tivozanib (Figure 4(a)). Notably, sitagliptin also did not enhance the impact of VEGF-A_165_ on the iBREC barrier even at higher concentrations (Figures 3 and 4).

### 3.3. Diprotin A Persistently Decreased the Cell Index of Unchallenged iBREC, but Did Not Change That of VEGF-A_165_-Exposed iBREC

**Figure 5:**
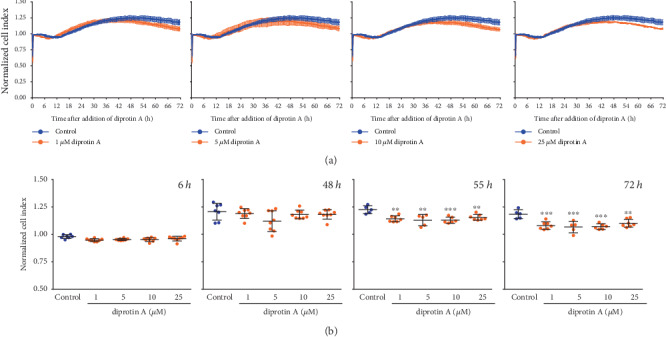
Prolonged treatment with diprotin A affected the barrier function of unchallenged iBREC. The CI was determined continuously during exposure of confluent iBREC to 1-25 *μ*M diprotin A. Similar to sitagliptin, this inhibitor of DPP-4 also induced a significant decline of the CI although this change was further delayed, starting about 55 h after its addition. (a) CI values, normalized in relation to those measured immediately before addition of diprotin A, are shown as means and standard deviations of data from at least five wells. (b) Statistical analyses of data from indicated time points after addition of diprotin A were performed as described in Materials and Methods. ^∗∗^*p* < 0.01 and ^∗∗∗^*p* < 0.001 compared to control.

**Figure 6:**
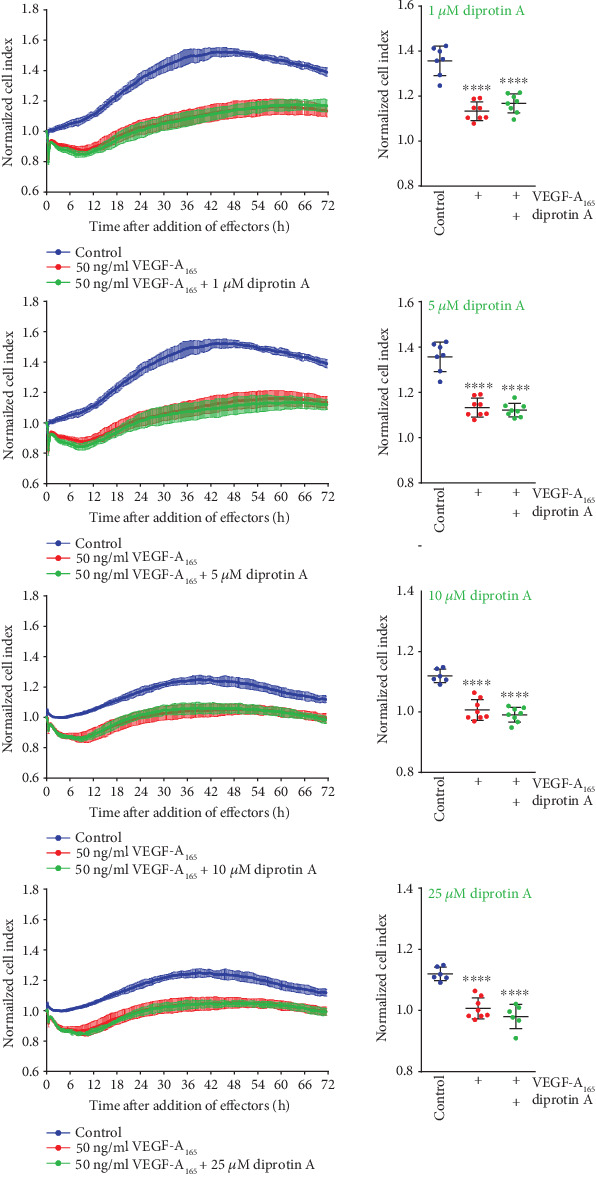
Diprotin A did not enhance the detrimental effect of VEGF-A on the iBREC barrier. Confluent iBREC were exposed to 1-25 *μ*M diprotin A and VEGF-A_165_, and the CI was determined continuously. Diprotin A did not influence the CI decrease caused by VEGF-A_165_. Normalized CI values are presented as means and standard deviations from at least six wells. Normalization and statistical analyses of data recorded 72 h after addition of diprotin A and VEGF-A_165_ were performed as described in Materials and Methods. ^∗∗∗∗^*p* < 0.0001 compared to control.

We also included the structurally unrelated (see Figure 1(a) for comparison) bacterial tripeptide Ile-Pro-Ile (diprotin A) in our studies to confirm that the observed effect of sitagliptin on the iBREC barrier was rather due to inhibition of the target DPP-4 and not an unspecific interaction of this molecule. Very similar to sitagliptin, diprotin A (final concentrations: 1-25 *μ*M) persistently reduced the CI of confluent iBREC in a dose-dependent manner, although this effect was delayed and reached statistical significance about 55 h after its addition (Figure 5). Diprotin A (final concentrations: 1-25 *μ*M) also did not modulate, prevent, or revert the VEGF-A_165_-induced decline of the CI (Figure 6, data not shown).

### 3.4. Sitagliptin and Diprotin A Stabilized the Expression of Proteins Involved in Paracellular Transport

**Figure 7:**
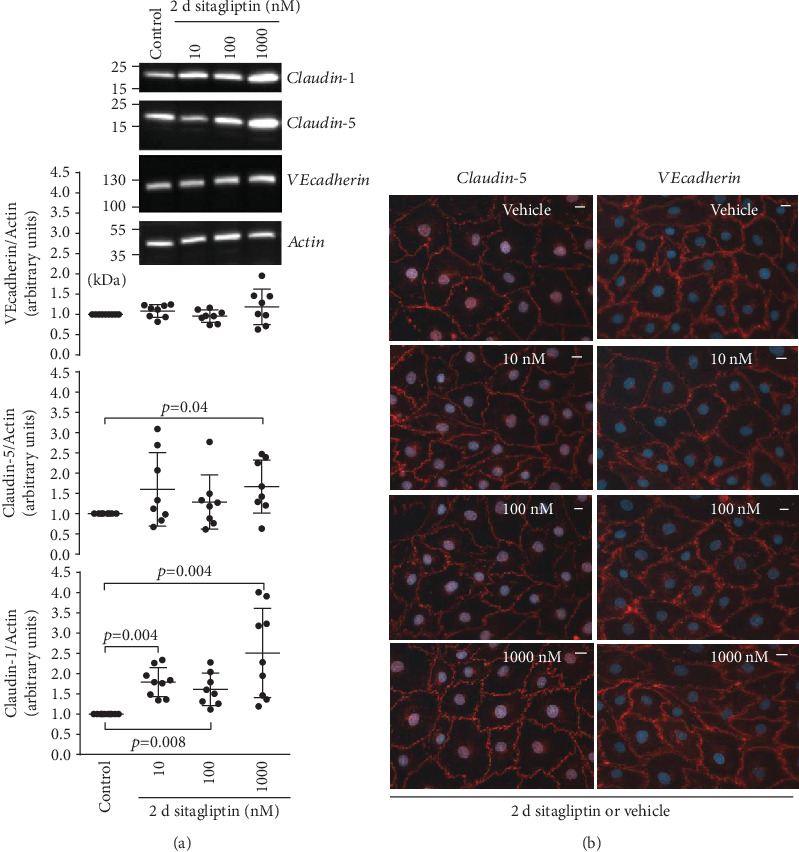
Prolonged treatment of iBREC with sitagliptin increased expression of claudin-1 and changed the plasma membrane localization of VEcadherin. (a) After exposure of confluent iBREC to 10-1000 nM sitagliptin for two days, cells were harvested for preparation of cell extracts, followed by Western blot analyses. Expression of claudin-1 was higher after treatment with sitagliptin at all concentrations used, but only 1 *μ*M sitagliptin led to a significantly higher level of claudin-5; expression of VEcadherin was not changed. Signals were normalized as described in Materials and Methods; *n* ≥ 8 for each condition. (b) Immunofluorescence staining showed that plasma membrane-localized claudin-5 was not affected by the inhibitor of DPP-4, but staining of VEcadherin at the plasma membrane appeared more diffuse when cells had been exposed to sitagliptin. Scale bar: 10 *μ*m.

**Figure 8:**
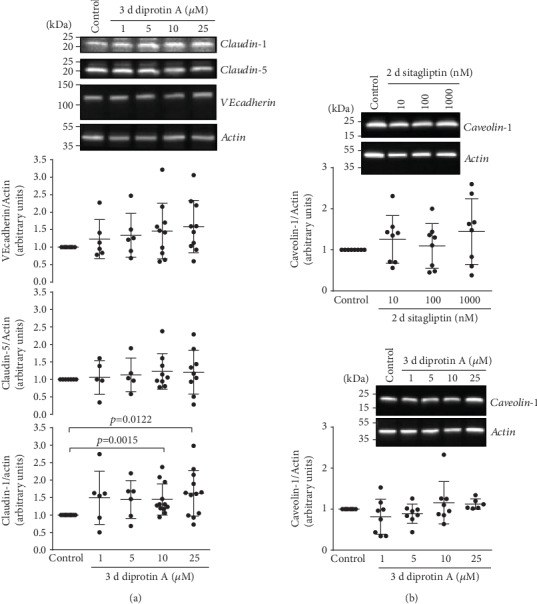
Prolonged treatment of iBREC with diprotin A increased expression of TJ protein claudin-1. After exposure of confluent iBREC to (a, b) 1-25 *μ*M diprotin A for three days or (b) 10-1000 nM sitagliptin for two days, cells were harvested for preparation of cell extracts, followed by Western blot analyses. (a) Expression of claudin-1 was significantly higher after treatment with 10 and 25 *μ*M diprotin A, but those of claudin-5 or VEcadherin were not changed. (b) Expression of caveolin-1 was also not affected by treatment with sitagliptin or diprotin A. Signals were normalized as described in Materials and Methods; *n* ≥ 6 for each condition.

Exposure of an iBREC monolayer to inhibitors of DPP-4 resulted in a decrease of the CI indicating a disturbance of the formed barrier (see Sections 3.1 and 3.1). Because such dysfunction might correlate with changes in the expression and/or subcellular localization of proteins involved in paracellular flow (i.e., TJ proteins claudin-1 and claudin-5 or AJ protein VEcadherin) or transcellular transport (i.e., caveolin), we assessed their expression by iBREC treated with 10-1000 nM sitagliptin for two days. Then, the CI was already low which is indicative of a nonfunctional barrier, and putative changes in the expression of proteins involved in barrier formation or stabilization should be evident. Western blot analyses of cell extracts, however, demonstrated that expressions of TJ proteins claudin-1 and claudin-5 were higher, although the changes were statistically significant only for claudin-1 (Figure 7(a)); expression of the AJ protein VEcadherin remained unchanged (Figure 7(a)). VEcadherin was also not subject to degradation or persistent posttranslational modification as indicated by a single, strong band at 130 kDa without any additional bands due to immunoreactive proteins of other mobility. To assess whether exposure to sitagliptin for two days might affect their subcellular localizations, we visualized the relevant proteins by immunofluorescence stainings. These showed a predominant localization of claudin-5 at the plasma membrane which appeared unchanged by treatment with sitagliptin (Figure 7(b)). The weak claudin-1-specific staining of the plasma membrane also remained stable during treatment with the DPP-4 inhibitor (data not shown). VEcadherin, however, was distributed more diffusely in the plasma membranes of cells exposed to sitagliptin, and this effect was more pronounced when higher concentrations of 100 nM or 1 *μ*M sitagliptin were used (Figure 7(b)). As the barrier-disturbing effect of diprotin A was significant from 55 h onwards after its addition, expression of candidate proteins was assessed after treating iBREC for three days with the inhibitor. Expression of claudin-1 was then higher, but those of claudin-5 and VEcadherin were not affected (Figure 8(a)). The observed decreased CI could also have been a consequence of an enhanced transcellular transport, but caveolin-1—a protein involved in this process—was stably expressed (Figure 8(b)).

### 3.5. Inhibitors of DPP-4 Lowered Expression or Changed Subcellular Localizations of Proteins Involved in Cell Adhesion

**Figure 9:**
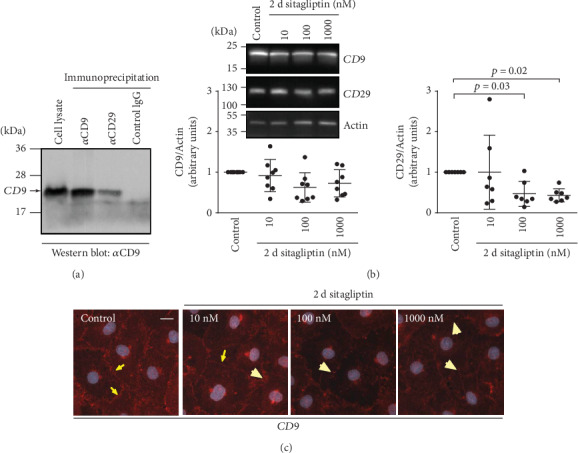
Prolonged treatment of iBREC with sitagliptin decreased expression of CD29 and changed the amount of plasma membrane-localized CD9. (a) Proteins were immunoprecipitated from whole cell extracts with antibodies specific for CD9 or CD29 and analyzed by Western blot with an antibody binding to CD9. Precipitates obtained with both antibodies (*α*CD9 and *α*CD29) contained the tetraspanin CD9 but not samples derived from precipitation with an isotype-matched control antibody, indicating that CD9 and CD29 are present in the same protein complex. (b) Confluent iBREC exposed to 10-1000 nM sitagliptin for two days were harvested for preparation of cell extracts followed by Western blot analyses. Expression of CD29 and CD9 was lower after treatment with sitagliptin, although the differences were statistically significant only for CD29. Signals were normalized as described in Materials and Methods. (c) Prominent CD9-specific staining was observed at the plasma membrane of control cells (yellow arrows). This staining was more diffuse and less intense (yellow arrowheads) after treatment of the cells with the DPP-4 inhibitor for two days. Scale bar: 10 *μ*m.

Because of the cells' direct contact with the electrodes, the lower CI could also have been a result of their impaired adhesion [29]. One obvious candidate protein mediating binding to fibronectin is the fibronectin receptor consisting of the subunits CD29/integrin *β*1 and CD49e/integrin *α*5, both of which are expressed by iBREC (Figure 9(b); data not shown) [30, 35]. Our previous studies also confirmed participation of the tetraspanin CD9 in adhesion of iBREC, and in various cell types, CD9 and CD29 are essential parts of adhesion complexes [30, 35, 36]. Indeed, a complex of CD29 with CD9 could be precipitated from protein extracts prepared from confluent iBREC (Figure 9(a)). In accordance with a potential involvement in sitagliptin-induced processes, treatment of iBREC with the DPP-4 inhibitor for two days resulted in a considerably lower expression of CD29, whereas amounts of CD9 were slightly reduced but without reaching statistical significance (Figure 9(b)). Interestingly, treatment with sitagliptin for two days resulted in a more diffuse and interrupted CD9-specific immunostaining of the plasma membrane (Figure 9(c)).

**Figure 10:**
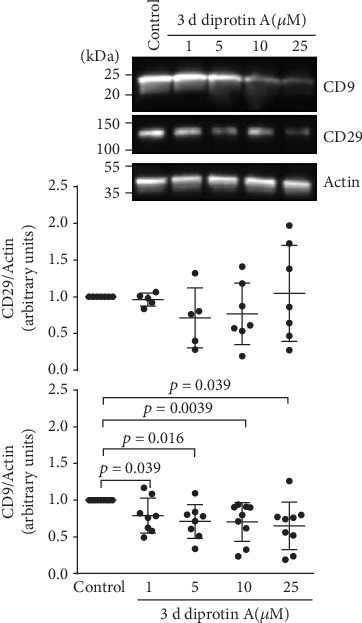
Prolonged treatment of iBREC with diprotin A decreased expression of CD9. After exposure of confluent iBREC to 1-25 *μ*M diprotin A for three days, cells were harvested for preparation of cell extracts to be analyzed by Western blot. Expression of CD9 was significantly lower after treatment with diprotin A. Signals were normalized as described in Materials and Methods; *n* ≥ 5 for each condition.

Exposure to diprotin A for three days significantly lowered the expression of CD9, while that of CD29 was partly, although not significantly, decreased (Figure 10).

Summarizing, both structurally unrelated inhibitors of DPP-4 induced a delayed but persistent decline of the CI, increased the expression of the TJ protein claudin-1, and lowered those of CD29 or CD9.

## 4. Discussion

To assess presumed damaging effects of DPP-4 inhibitors on the inner blood-retina barrier, we investigated whether sitagliptin or diprotin A changed the cell index as a measure of barrier function of immortalized microvascular endothelial cells of the bovine retina. In addition, potentially affected expression and subcellular localization of proteins involved in the regulation of paracellular flow, transcellular transport, or adhesion to the extracellular matrix were analyzed. Although limitations of the model system have to be considered, the observed changes induced by prolonged exposure to sitagliptin at reasonable concentrations nicely mimic long-term treatment of diabetes patients with such drugs.

Because sitagliptin induced only small alterations of TEER with unclear clinical relevance, we performed more sensitive continuous cell index measurements to detect even subtle and transient changes of the iBREC barrier function. Indeed, both inhibitors of DPP-4 investigated significantly and strongly decreased the cell index in a nearly concentration-dependent manner. This effect was evident about two days after their addition, and it was clearly visible when concentrations close to their published IC_50_ values were used in the experiments (Figures 2 and 5). In general, measuring the TEER is a reliable technique to study the integrity of barriers of endothelial or epithelial cell monolayers, and lower TEER values result from increased paracellular and/or transcellular flow [32]. Indeed, persistent barrier dysfunction of an iBREC monolayer due to disorganized TJs induced by exposure to VEGF-A_165_ can easily be determined with a volt-ohm meter and hand-held electrodes [18, 21]. Since such measurements are discontinuous and performed outside the incubator, they are sensitive to environmental factors, i.e., temperature or pH fluctuations, and subtle or transient effector-dependent TEER changes might be overlooked [33]. In contrast, continuous determination of the cell index of cells cultivated on gold electrodes is less prone to any environmental impact because the cells remain in the incubator; even subtle and transient changes due to impaired paracellular and transcellular flow are therefore easily detected [22, 27, 28]. The nature of the cells' adhesion—in addition to paracellular and transcellular flow—codetermines the measured cell index values because of direct attachment of the cells to the electrodes [27, 29]. The apparently inconsistent findings concerning the effect of sitagliptin on the iBREC barrier might be due to interference of the DPP-4 inhibitor with cell adhesion (affecting the cell index but not the TEER), which is discussed in detail below.

As both inhibitors have distinct and unrelated structures (Figure 1(a)), it seems very unlikely that their similar effects are due to unspecific interactions, indicating that the decreased cell index indeed is very likely a consequence of DPP-4 inhibition. In contrast to results from experiments with macrovascular EC originating from veins in which both inhibitors of DPP-4 induced an increase of permeability within thirty minutes, the barrier of microvascular iBREC was impervious to such short-term treatment, in accordance with similar behavior of primary BREC [10, 12]. It is important to point out that even subtle effects on barrier integrity would have been detected by the sensitive cell index measurements [22, 28]. These apparently contradictive findings most likely reflect the fundamentally different behavior of EC originating from different vascular beds determined by their distinct gene expression patterns, again underlining the importance of correct selection of suitable in vitro models [37]. Concerning investigations of potentially disturbing effects on the cells of the inner BRB, primary or immortalized microvascular BREC are clearly a more appropriate model than macrovascular HUVEC.

Extended inhibition of DPP-4 for two or three days stabilized or even increased the expression of VEcadherin, claudin-5, and claudin-1 by iBREC (Figure 7). Since these proteins are involved in the limitation of paracellular flow in microvascular REC, this finding is somewhat surprising [12, 18–22, 38]. It can be concluded that the low cell index observed during treatment with DPP-4 inhibitors was most likely not due to an increased paracellular flow. On the other hand, it is a plausible assumption that the impaired adhesion of iBREC to the extracellular matrix, namely, fibronectin, might have led to the observed decline of the cell index. Such changes of adhesion properties are recognized by impedance measurements of cells grown on gold electrodes [28, 29]. Proteins likely involved are not only the fibronectin receptor (⟶ subunit CD29/integrin *β*1) but also the tetraspanin CD9 because binding of a specific antibody to this protein hinders adhesion of iBREC to fibronectin-coated surfaces [30]. Indeed, not only was a considerable portion of CD9 relocated from the plasma membrane after treating the cells with sitagliptin for two days, but expression of CD9 or CD29 was also lower after extended exposure of iBREC to DPP-4 inhibitors. As CD9 and CD29 also act as components of the same protein complex—proven by immunoprecipitation—reduction of both proteins in the plasma membrane might synergistically result in an impaired adhesion of the cells, associated with a lower cell index. Considering the effect on CD9, it is of particular interest that this tetraspanin plays a role in the interaction of EC with leucocytes in inflammatory processes, and one could therefore speculate that its depletion from the plasma membrane of retinal endothelial cells induced by sitagliptin might be at least in part also beneficial [39, 40]. Whether or not prevention of inflammation in the retina of type 1 diabetic rats by sitagliptin is due to this process remains unresolved, although it definitely is an exciting speculation [11]. At least the observation that the inhibitor of DPP-4 linagliptin reduces the number of monocytes adhering to huREC in vitro supports this hypothesis [13]. Similar to the previous observation that the elevated permeability of primary BREC induced by TNF*α* was not prevented by sitagliptin, inhibition of DPP-4 did not also block or revert the VEGF-A_165_-induced decline of the CI of iBREC (Figures 3, 4, and 6) although the signal transduction pathways of both growth factors likely differ in detail in this cell type: blocking of phosphoinositol-3 kinase or treatment with dexamethasone prevents TNF*α*- but not VEGF-A-induced barrier dysfunction; exposure to TNF*α* results in the loss of TJ protein claudin-5, whereas VEGF-A_165_ slightly enhances its expression and rather lowers that of claudin-1 [12, 18, 19, 22, 38].

Extended inhibition of DPP-4 resulted in a persistent barrier dysfunction of microvascular REC in vitro in an order similar to that caused by permeability-inducing factor VEGF-A_165_. In view of these data, it cannot be ruled out that prolonged exposure of the retinal vasculature to the systemically applied drug might result in a disturbance of the inner blood-retina barrier contributing to DME in vivo. This is an important observation with therapeutic consequences as an edema potentially induced by sitagliptin or another DPP-4 inhibitor would likely not or only weakly respond to standard treatment with VEGF-A-binding proteins like ranibizumab or aflibercept.

## 5. Conclusion

Based on our data obtained with a well-established in vitro model, we conclude that the prolonged inhibition of DPP-4 destabilizes the barrier formed by microvascular REC. DPP-4 inhibitors like sitagliptin will also most likely not be able to prevent or revert the VEGF-A_165_-induced barrier dysfunction frequently observed in DME.

## Figures and Tables

**Table 1:** Primary and secondary antibodies used.

Target	Host and type	Source	Working concentrations
Actin	Mouse, monoclonal	Clone AC-40, Abcam (Cambridge, United Kingdom), ab11003	WB: 500 ng/ml
	Mouse, monoclonal	Clone 5J11, Abcam, ab190301 or Bio-Techne (Wiesbaden, Germany) NBP2-25142	WB: 700 ng/ml
Caveolin-1	Rabbit, polyclonal	Abcam, ab2910	WB: 20 ng/ml IF: 2 *μ*g/ml
CD9 (tetraspanin-29)	Mouse, monoclonal, IgG_2_	Clone IVA50, EXBIO (Vestec, Czech Republic, via BIOZOL, Munich, Germany), 11-354C100	WB: 40 ng/ml IF: 10 *μ*g/ml IP: 1 *μ*g/ml
CD29 (integrin *β*1)	Mouse, monoclonal, IgG_1_	TS2/16, Thermo Fisher Scientific (Langenselbold, Germany), 14-0299-82	WB: 170 ng/ml IF: 4 *μ*g/ml IP: 1 *μ*g/ml
Claudin-1	Rabbit, polyclonal	JAY.8, Thermo Fisher Scientific, 51-9000	WB: 1 *μ*g/ml
Claudin-1	Rabbit, polyclonal	Aviva Systems Biology (BIOZOL, Eching, Germany), ARP33623_P50	IF: 4 *μ*g/ml
Claudin-5	Rabbit, polyclonal	Z43.JK, Thermo Fisher Scientific, 34-1600	WB: 100 ng/ml IF: 2.5 *μ*g/ml
VEcadherin	Rabbit, polyclonal	Cell Signaling Technology B.V. (Frankfurt, Germany), 2158S	WB: 1 : 1000 IF: 1 : 100
Control IgG UNLB-IgG	Mouse, monoclonal, IgG_1_	Clone 15H6, Southern Biotech (Birmingham, United Kingdom)	IP: 1 *μ*g/ml
Whole IgG, rabbit	Goat, polyclonal, coupled to HRP	Bio-Rad (Munich), 170-5046	WB: 1 : 30000
Whole IgG, mouse	Goat, polyclonal, coupled to HRP	Bio-Rad, 170-5047	WB: 1 : 30000

IF: immunofluorescence; IP: immunoprecipitation; WB: Western blot.

**Table 2:** Characteristics of inhibitors used.

Inhibitor	Targeted protein	IC_50_	Final concentrations	References	Provider^a^
Sitagliptin	DPP-4	18 nM	10-1000 nM	[4]	Selleckchem
	DPP-2/QPP	100 *μ*M			
	DPP-8	48 *μ*M			
	DPP-9	100 *μ*M			

Diprotin A (Ile-Pro-Ile)	DPP-4	3 *μ*M	1-25 *μ*M	[31]	Tocris
	Other peptidases	>100 *μ*M			

Tivozanib (KRN951, AV-951)	VEGF receptor 1	30 nM	10 nM	[41]	Selleckchem
	VEGF receptor 2	6.5 nM			
	VEGF receptor 3	15 nM			
	PDGF receptor *α*	40 nM			
	PDGF receptor *β*	49 nM			

^a^Selleckchem: Selleckchem via Absource GmbH, Munich; Tocris: Tocris via Bio-Techne. DPP: dipeptidyl peptidase; QPP: quiescent cell proline dipeptidase.

## Data Availability

The data supporting the findings of this study are available through OPEN ACCESS, as well as from the corresponding author upon request.
